# Joint Iterative Fast Projection Matching for Fully Automatic Marker-free Alignment of Nano-tomography Reconstructions

**DOI:** 10.1038/s41598-020-62949-1

**Published:** 2020-04-30

**Authors:** Chun-Chieh Wang

**Affiliations:** National Synchrotron Radiation Research Center, 30076 Hsinchu, Taiwan

**Keywords:** Mathematics and computing, Software, Computational biology and bioinformatics, Image processing

## Abstract

Highly accurate, fully automatic marker-free image alignment plays an important role in nano-tomographic reconstruction, particularly in cases where the spatial resolution of the tomographic system is on the nanometer scale. However, highly accurate marker-free methods such as the projection matching method are computationally complex and time-consuming. Achieving alignment accuracy with reduced computational complexity remains a challenging problem. In this study, we propose an efficient method to achieve marker-free fully automatic alignment. Our method implements three main alignment procedures. First, the frequency-domain common line alignment method is used to correct the in-plane rotational errors of each projection. Second, real-space common line alignment method is used to correct the vertical errors of the projections. Finally, a single layer joint-iterative reconstruction and re-projection method is used to correct the horizontal projection errors. This combined alignment approach significantly reduces the computational complexity of the classical projection matching method, and increases the rate of convergence towards determining the accurate alignment. The total processing time can be reduced by up to 4 orders of magnitude as compared to the classical projection matching method. This suggests that the algorithm can be used to process image alignment of nano-tomographic reconstructions on a conventional personal computer in a reasonable time-frame.

## Introduction

Image alignment is an important procedure that is performed prior to image reconstruction in 3D computed tomography. This step is essential as the precision of alignment affects the spatial resolution of the tomographic reconstructions. Although various image alignment methods have been proposed to date, the majority of these techniques necessitate the placement of additional high-contrast markers as reference points on the specimen being imaged. This facilitates the manual or automatic alignment of the projections obtained from different tilt angles with high precision using fiducial markers^1–5^. However, the added markers may contaminate specimens, and the removal of these markers from the tomographic reconstructions requires additional post-processing that might not be straightforward. To achieve automatic alignment, the runout error of the sample rotational stage can be fine-corrected by using a high-precision detection and compensation hardware system^6^. However, this method is not useful for correcting errors that are generated from non-reproducible sources, for example, the thermal variation of the sample or substrate that results from the heating of high-flux illumination sources like X-rays or electrons. Alternately, marker-free image alignment methods such as the cross-correlation^7,8^, feature matching^1,9–11^, and projection matching methods^12–14^ tend to be more suitable for high-resolution tomography applications. However, the alignment accuracy is strongly dependent on the computational complexity or processing time of the methods listed above. In brief, the cross-correlation method finds the best positional correlation coefficient between two adjacent sequential projections and has the fastest processing time. However, this method has the least alignment accuracy as it does not take the rotational relationship between the adjacent projections into account. On the contrary, the feature matching or projection matching methods provide a highly accurate estimate of the rotational relationship between adjacent projections. However, a large number of positional fittings and/or iterative calculations are required in these methods, leading to increased computational complexity. The classical projection matching method in particular cannot be processed on a conventional personal computer due to the high computational complexity. A major efficiency limitation of classical projection matching method is that the entire 3D tomography dataset has to be reconstructed in order to generate the subsequent 2D re-projections, which is an extremely time-consuming process. For example, entire temporary 3D tomography datasets are reconstructed using iterative algorithms such as the maximum-likelihood estimation method (ML-EM)^15^ or the simultaneous iterative reconstruction technique (SIRT)^16^ in the classical projection matching method. The use of iterative procedures in the classical projection matching method presents itself as the main bottle neck in the achievable processing speed.

An efficient image alignment algorithm based on the simplified projection matching method, which is known as fast projection matching or Faproma, has been proposed by Wang *et al*.^17^ to reduce the computational complexity and preserve the alignment accuracy of the classical projection matching method. The Faproma method is a combination of the common-line^18,19^ and projection matching methods. The common-line method addresses the errors associated with the vertical shift and in-plane rotation. Tilt errors can be effectively corrected using the projection matching method. In Faproma, single layer projection matching can be used to correct for any residual horizontal errors in each projection if the vertical and in-plane rotational errors have already been corrected using the common-line method. This indicates that the temporary 3D reconstruction process in classical projection matching method can be reduced to a temporary 2D reconstruction process in each of the projection matching iterations. This is the key concept behind the Faproma method that can significantly reduce the computational complexity or total processing time of the classical projection matching method.

The joint iterative reconstruction and re-projection method (JIRRM) has been proposed by Gürsoy *et al*.^20^ to significantly reduce the number of reconstruction iterations required by the classical projection matching method. In the JIRRM, the ML-EM/SIRT reconstruction processed only once iteration during each of iterations of classical projection matching algorithm, as opposed to being run multiple times in the classical projection matching method. This shows that both iterative reconstruction and iterative projection matching are processed in parallel in the JIRRM algorithm.

In this study, we propose a more efficient projection matching-based image alignment method that integrates the JIRRM in the Faproma algorithm to further reduce the computational complexity in the single layer projection matching iterations of the Faproma. We demonstrate that the joint iterative fast projection matching (JI-Faproma) method not only significantly speeds-up the alignment process, but also preserves the spatial resolution of the tomographic reconstructions along three dimensions in X-ray nano-tomography.

## Methods

**Figure 1:**
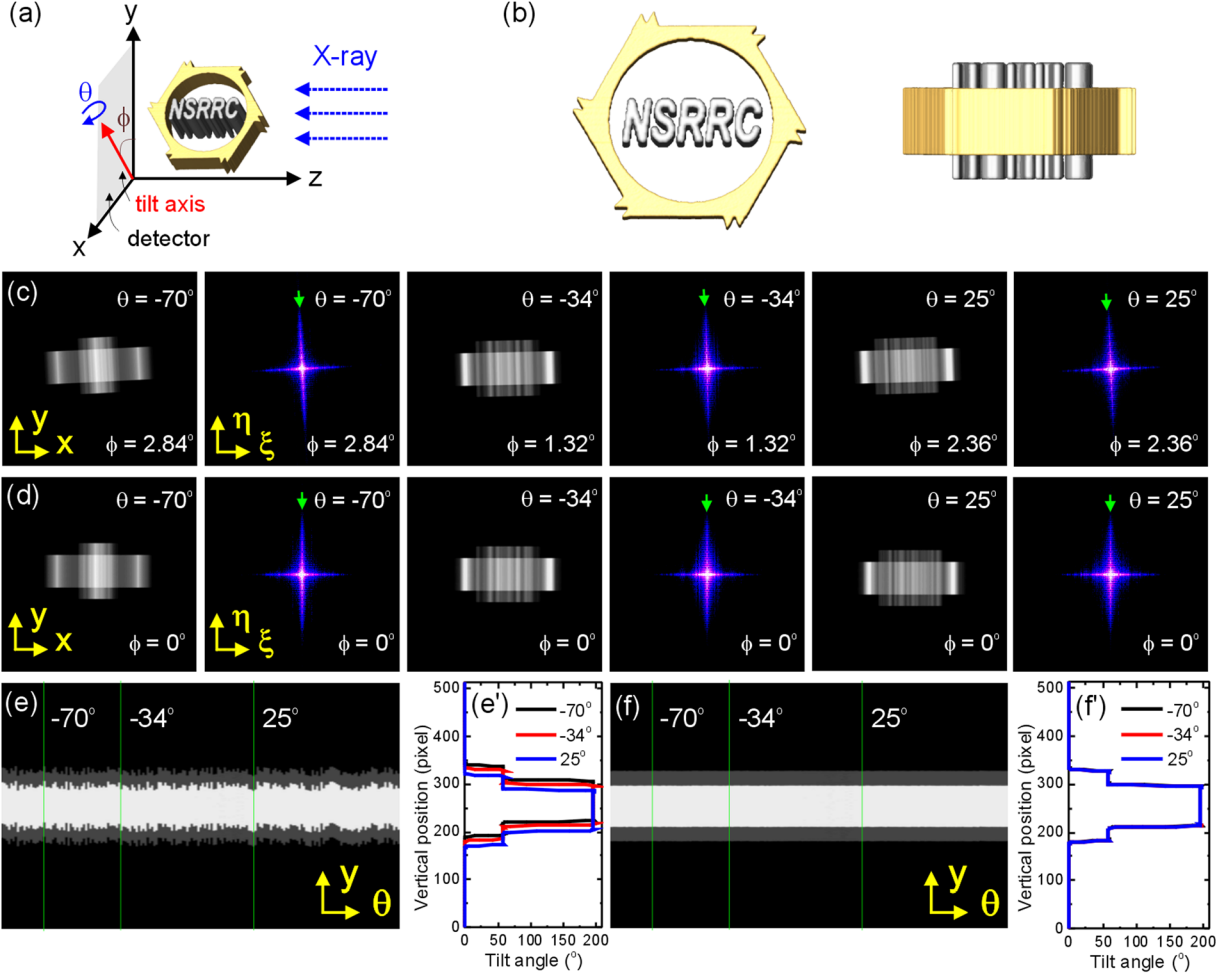
In-plane rotational and vertical errors corrected by using common-line method. (**a**). The coordination diagram. (**b**). Computer-generated 3D phantom. (**c**) Projections of the 3D phantom simulated from different tilt angles and their corresponding 2D Fourier transforms with random errors in the *ϕ*, *y*, and *x* dimensions. (**d**) In-plane rotational and vertical shifts corrected projections and their corresponding 2D Fourier transforms. Green arrows indicate the common lines in frequency domain. (**e**) Real-space common lines with random errors in the *y* axis obtained from different tilt angles (*θ*). (**f**) Corrected real-space common lines obtained from different tilt angles. (e’) and (f’) show the line profiles of the raw and vertical-error-corrected real-space common lines obtained at tilt angles of −70°, −34°, and 25°, respectively.

Figure 1(a) shows the coordination diagram used in this study and Fig. 1(b) shows different views of the computer-generated 3D phantom that was used for evaluating the alignment performance of the JI-Faproma method. Each projection requires the correction of three spatial errors namely, the in-plane rotational error Δ*ϕ*, vertical error Δ*y*, and horizontal error Δ*x*. The procedures in the JI-Faproma algorithm used to correct the three spatial errors in each projection are given by (1) the common-line method operating in the frequency domain for the correction of in-plane rotational errors, (2) the common-line method operating in the real space for the correction of vertical errors, and (3) the single layer JIRRM method for the correction of horizontal errors. The alignment accuracy of the JI-Faproma method was evaluated on a series of projection images obtained using the ideal 3D phantom shown in Fig. 1(b), with random errors in the *ϕ*, *y*, and *x* dimensions (as shown in Fig. 1(c) and Supplementary Movie S1).

### In-plane rotational error correction

The first step in the JI-Faproma method involves the correction of the in-plane rotational errors in the raw projection images using the common-line method. In the common-line method, if the raw projections are well-aligned along all three dimensions in the real space, the central line frequency spectrums of all projections tend to be aligned along the tilt axis in the frequency domain^18^. This common central spectrum along the tilt axis is referred to as the common line. Figure 1(c) shows a few simulated raw projections and their corresponding 2D Fourier transforms, with random errors in three dimensions, generated from tilt angles (*θ*) of −70°, −34°, and 25°. Figure 1(d) shows the results from the in-plane rotational error correction for the simulated data shown in Fig. 1(c). The in-plane rotational errors can be fine-corrected by tilting the common line of each projection image (indicated by the green arrows in Fig. 1(c),(d) along the vertical tilt axis).

### Vertical error correction

The second step in the JI-Faproma method involves the correction of the vertical offset of each projection using the common-line method in real space. Once the in-plane rotational errors have been corrected, the integrated intensity distributions, which are obtained by integrating along the *x*-axis (Fig. 1(a)) of each projection corresponding to the tilt angle, must be identical. This generates the common-line, which is essentially the Fourier transform of the intensity distribution of each projection under the common integral. Figure 1(e),(f) show the raw and vertical shift corrected integrated intensity distributions obtained at different tilt angles. Figure 1(e’),(f’) show the line profiles of the raw and vertical shift corrected integrated intensity distributions obtained at tilt angles of −70°, −34°, and 25°, respectively. Both differential and iterative methods are used in the second step of the JI-Faproma algorithm in order to eliminate the alignment errors resulting from any inhomogeneity and variation in the background of each projection. The differential and iterative alignment equations for vertical error correction can be defined as:

1 $$\mathop{\varDelta y}\limits^{{argmin}}\Vert \frac{\partial {\int }_{R}{\tilde{p}}_{{\theta }_{i}(f)(x,y-\varDelta y)dx}}{\partial y}-\frac{1}{k}\mathop{\sum }\limits_{l=1}^{k}\frac{\partial {\int }_{R}{\tilde{p}}_{{\theta }_{i}(f)(x,Y)dx}}{\partial Y}\Vert ,$$

where *θ* is the tilt angle, *f* is the 3D density function of the object, *k* is the number of projections, and Δ*y* is the vertical shift correction corresponding to the raw projections at different tilt angles (*θι*). The second averaged term in Eq. (1) serves as a reference for the vertical alignment of the raw differential integrated intensity profiles. A temporary averaged differential integrated intensity profile, which is calculated from the average of the newly aligned differential integrated intensity profiles, obtained using different tilt angles, serves as the new reference for the next iteration. The algorithm is iterated until both the temporary and new reference profiles are almost equal.

### Horizontal error correction

**Figure 2:**
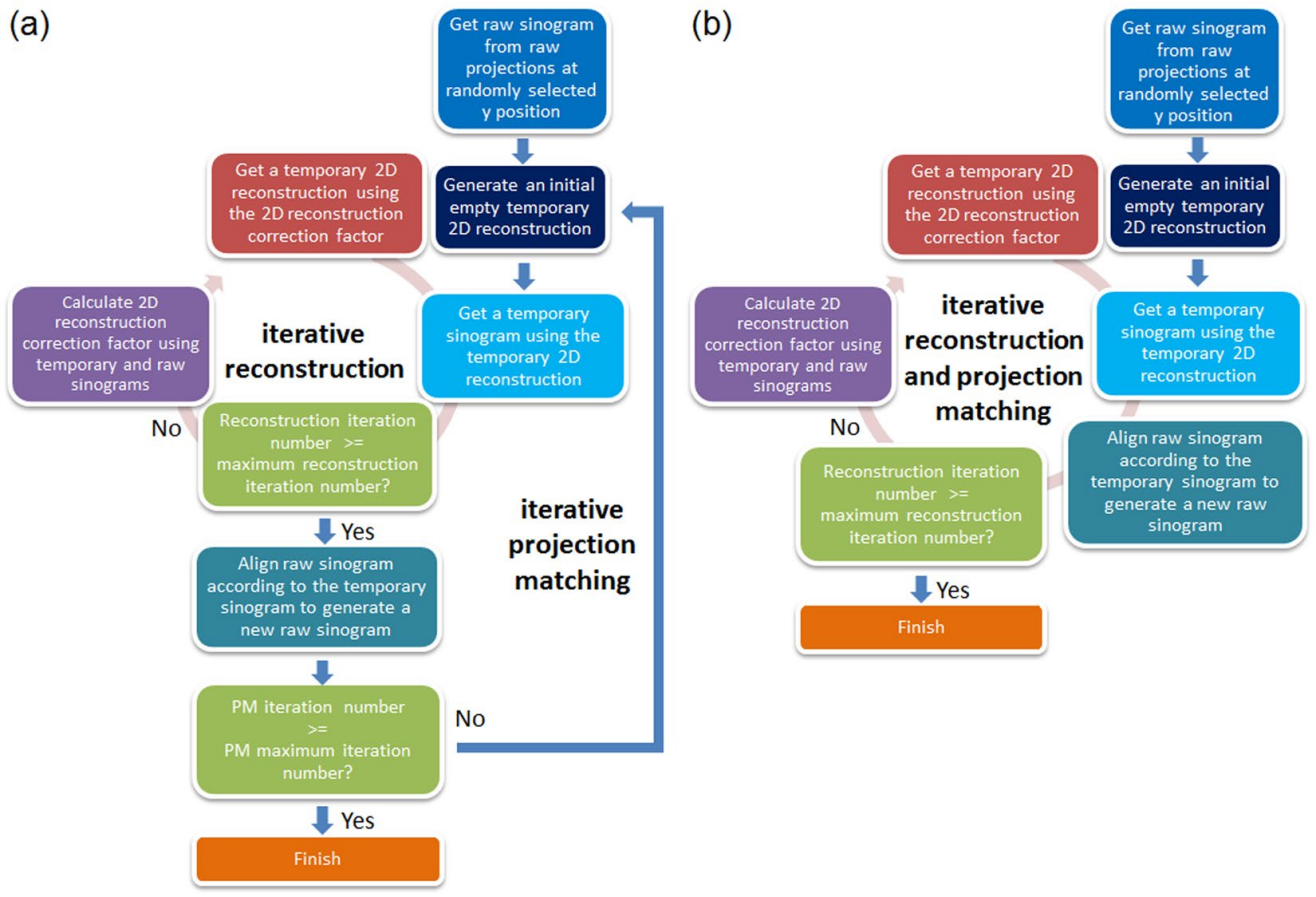
Flowchart comparison between the (**a**) Faproma, and (**b**) JI-Faproma for horizontal error correction.

The final step in the JI-Faproma method involves the correction of the horizontal error of each projection. Similar to the Faproma method, the horizontal alignment along only one randomly selected layer (parallel to the *x-z* plane, see Fig. 1(a)) is considered following the corrections of the vertical and in-plane rotational errors of each projection. The objective is to align raw sinogram according to the rotational relationship of each feature between the tilt angles in the selected reconstruction layer. In this study, the JIRRM was used to reduce the iterative procedure of single-layer projection matching in the Faproma algorithm, which is able to speed-up convergence. Figure 2 shows the flow charts of the Faproma and JI-Faproma methods. In the Faproma method, the iterative reconstruction is processed within each iterative projection matching step, which is the basic premise of the classical projection matching method. On the contrary, both the iterative reconstruction and iterative projection matching are processed together in the JI-Faproma algorithm to highly reduce the number of iterations required to approach convergence, which is the basic premise of the JIRRM method.

The first step of the horizontal shift correction process, referred to as the single layer JIRRM, is shown in Fig. 2(b). The one-dimensional cross-correlation method, which serves as the initial raw sinogram, is used to pre-align the raw sinogram corresponding to the randomly selected *y* layer. An initial empty temporary 2D reconstruction is then generated, similar to the first step in the ML-EM reconstruction^15^. The temporary 2D reconstruction is used to obtain a temporary sinogram, which is subsequently used to align and generate a new raw sinogram. The algorithm is terminated when the single layer JIRRM iteration number has reached the settled value. If the algorithm has not be terminated, the 2D reconstruction correction factor *A*(*i*,*j*) should be calculated from the temporary and raw sinograms using the following equations:

2 $$C{(i,j)}_{\theta }=\frac{RS{(j)}_{\theta }}{TS{(j)}_{\theta }},$$

3 $$\begin{array}{c}A(i,\,j)=\frac{1}{n}{\sum }_{\theta }R(\theta )\,{\rm{C}}\,{({\rm{i}},j)}_{\theta }\end{array}$$

where *TS* and *RS* represent the temporary and raw sinograms, respectively, *n* is the projection number, *θ* is the azimuthal angle of the projection, and *R* is the rotational matrix. The temporary 2D reconstruction is then multiplied by *A*(*i,j*) to generate a new temporary 2D image for the next iteration of the single layer JIRRM.

**Figure 3:**
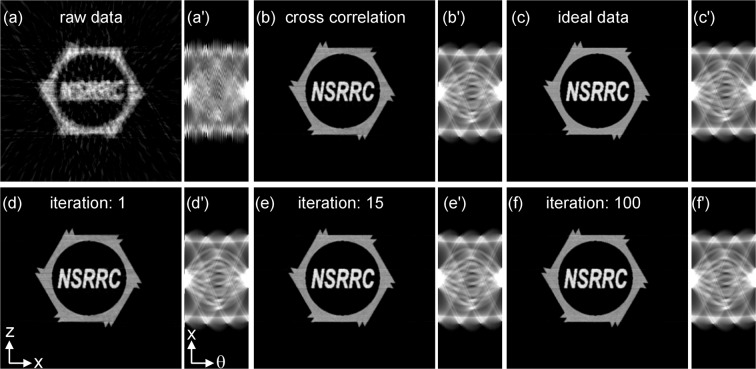
Simulation results of the JI-Faproma method. (**a**) 2D reconstruction by the raw sinogram, which was acquired at the randomly selected *y*-position of the raw projections. (**b**) 2D reconstruction after sinogram alignment by the cross-correlation method. (**c**) 2D reconstruction by using the ideal sinogram. 3(**d–f**) show the 2D reconstruction quality after using the JI-Faproma alignment method for 1, 15, and 100 iterations, respectively. (a’-f’) are the corresponding sinograms of the (**a–f**) 2D reconstructions, respectively.

Figure 3 shows the simulation results obtained using the JI-Faproma method. Figure 3(a) shows the 2D reconstruction of the raw sinogram (also shown in Fig. 3(a’)), which was acquired at the selected *y*-layer of the raw projections. Figure 3(b),(b’) show the reconstruction and sinogram alignment results from the simple cross-correlation method. Figure 3(c),(c’) show the ideal 2D reconstruction and perfectly aligned sinogram of the simulated 3D phantom, respectively. Figure 3(d–f),(d’–f’) show the image reconstruction and sinogram alignment results obtained using the JI-Faproma method for 1, 15, and 100 iterations, respectively. The reconstruction quality and sinogram alignment accuracy after 15 iterations of the JI-Faproma method (shown in Fig. 3(e) and 3(e’)) are comparable to the results from the ideal case (shown in Fig. 3(c),(c’)). Thus, this demonstrates that the JI-Faproma method is able to achieve fast convergence and highly accurate alignment in 3D tomography. The fine-aligned projections and their corresponding 2D Fourier transforms after the alignment of JI-Faproma method are shown in Supplementary Movie S2.

## Results

### Alignment accuracy vs noise level

**Figure 4:**
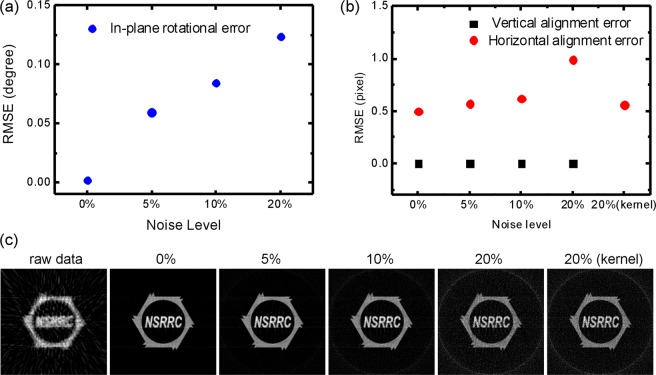
Performance of the JI-Faproma evaluated by comparing the alignment accuracy under different noise levels. (**a**) Root-mean-square error of the in-plane rotational error correction. (**b**) Root-mean-square errors of the vertical and horizontal error corrections. (**c**) Reconstruction quality after JI-Faproma alignment for test phantoms containing different levels of noise.

The performance of the JI-Faproma method was evaluated by comparing the alignment accuracy for in-plane rotational, vertical, and horizontal alignment errors under different noise levels. As shown in Fig. 4(a),(b), the corrections applied to both the in-plane rotational and vertical errors result in perfectly aligned projections/reconstructions when the test phantoms are noise-free. There is a significant increase in the root-mean-square error (RMSE) of in-plane rotational and horizontal alignments with increasing noise level in the test phantom. However, the in-plane rotational, vertical, and horizontal alignment errors are acceptable and within 0.12°, 0 pixel, and 1 pixel, respectively, for noise levels of up to 20%. A conventional method, such as a kernel filter, can be applied to decrease noise level of the images with low signal-to-noise ratio before the alignment. As shown in Fig. 4(b), a 3 × 3 kernel filter applied to the test phantom with a 20% noise level resulted in a decrease in the RMSE of the horizontal alignment from 1 to 0.6 pixels. Figure 4(c) shows the image reconstruction quality for test phantoms containing different levels of noise. In JI-Faproma, another strategy for very low signal-to-noise ratio and very low contrast level datasets is that high-contrast fiducial markers can be used as well for increasing both signal-to-noise ratio and contrast level of the projection datasets for the increase of alignment accuracy.

**Figure 5:**
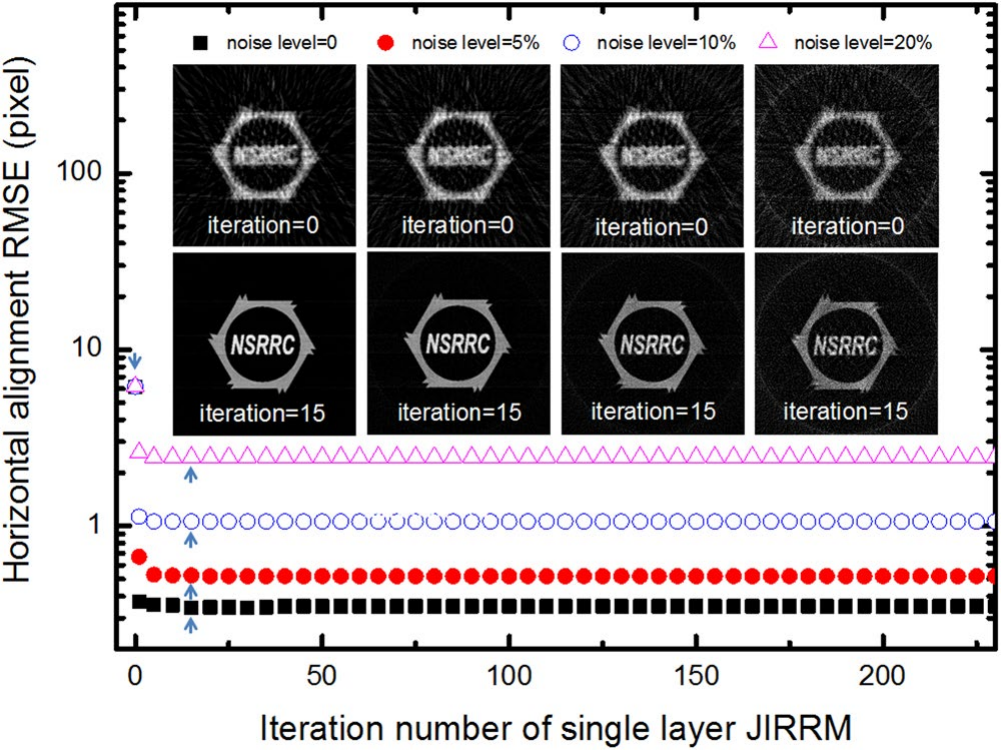
Ji-Faproma algorithm convergence under different noise level of raw projections.

Figure 5 shows the convergence of the JI-Faproma algorithm, wherein the RMSEs for different noise levels rapidly converged to their optimal value using less than 15 iterations of the single layer JIRRM method (indicated by blue arrows). The corresponding reconstructions for different noise levels and the number of iterations per reconstruction are also shown in Fig. 5.

### Comparison between JI-Faproma and Faproma

**Figure 6:**
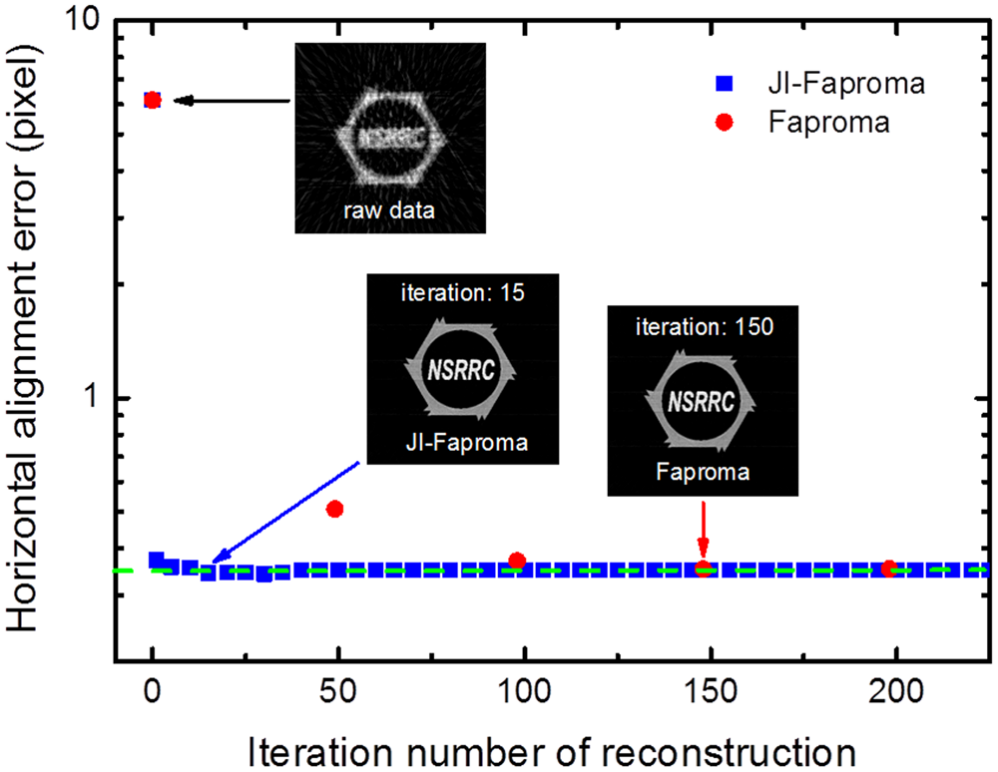
Comparison between the JI-Faproma and Faproma algorithms for variations in the horizontal alignment RMSE with increasing iteration number of reconstruction. Green-dashed line is close to the value of the minimum horizontal alignment errors both in Faproma and JI-Faproma.

The JI-Faproma method offers a higher processing speed for horizontal error correction as compared to the Faproma method. Figure 6 shows a comparison between the JI-Faproma and Faproma algorithms for variations in the horizontal alignment RMSE with increasing iteration number. In the Faproma method, a temporary sinogram, which is extracted from a temporary 2D reconstruction obtained via iterative reconstruction method, is used to align the raw sinogram. The Faproma algorithm typically requires 50 iterations for the ML-EM iterative reconstruction to reach convergent reconstruction quality. Figure 6 shows that the single layer JIRRM requires only 15 iterations of the ML-EM reconstruction to arrive at the minimum value of the horizontal alignment RMSE. On the other hand, the horizontal alignment RMSE reaches its minimum value after 3 iterations of the Faproma horizontal correction algorithm (single layer projection matching method), which is the equivalent of running the ML-EM reconstruction with 150 iterations.

### Alignment accuracy evaluation in a real nano-tomography case

The quality of 3D tomographic reconstruction depends on the alignment accuracy of the tomographic projections. This, in particular, has important implications in nano-tomography, which involves the use of electron tomography or X-ray nano-tomography systems. In this study, we demonstrate the alignment accuracy achieved by the JI-Faproma method in an X-ray nano-tomographic image dataset, which was obtained using synchrotron transmission X-ray microscopy (TXM) at the Taiwan Light Source (TLS). The TXM, which is housed at the BL01B1 beamline in the TLS (NSRRC, Taiwan), facilitates 2D radiography and 3D tomography acquisitions of specimens with a 60-nm spatial resolution using a Fresnel zone plate objective^21^. A Zernike phase ring is installed at the rear focal plane of the zone plate for phase contrast imaging, which is particularly useful for imaging materials composed of light elements. The TXM was used to acquire a series of high-resolution images of a diatom microfossil for tilt angles ranging from −85° to 85° with a 1° increment. Each image has a size of 512 × 512 pixels, and a pixel size of 30 × 30 nm^2^. The diatoms can be considered as pure phase objects in the hard X-ray region due to their high silica composition, and can be observed/visualized under the phase contrast mode of the TXM.

**Figure 7:**
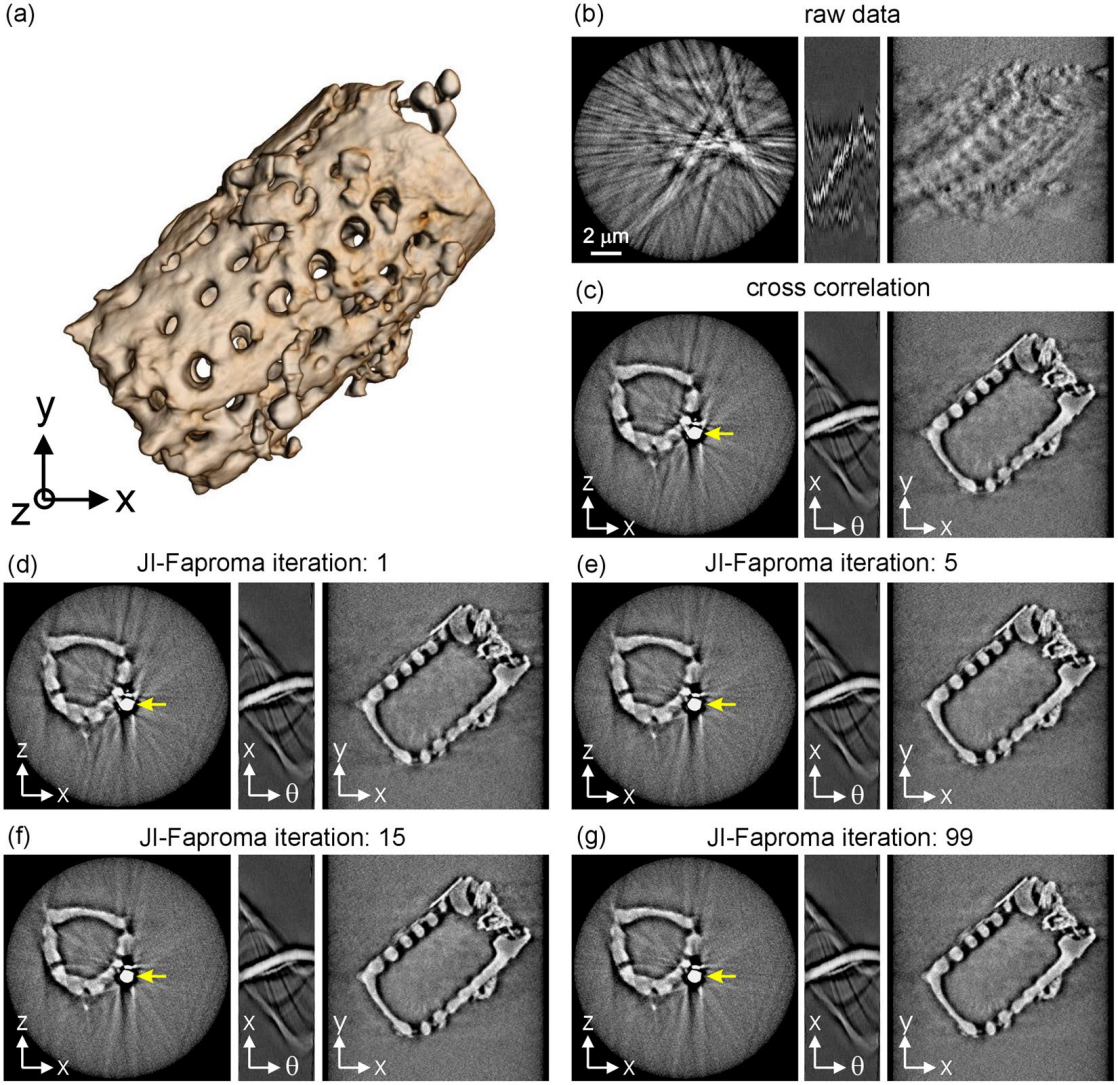
Alignment accuracy evaluation in a real X-ray nano-tomography case. (**a**) 3D tomography of a diatom microfossil, with image alignment performed using the JI-Faproma method. *x*-*z* and *x*-*y* plane cross-sections and *x-z* cross-section sinogram of the diatom 3D tomography, which were reconstructed (**b**) from the TXM raw data, (**c**) after the cross-correlation alignment, (**d–g**) after the different iteration number of JI-Faproma alignments.

Figure 7(a) shows the 3D reconstruction of the diatom, with image alignment performed using the JI-Faproma method. Figure 7(b) shows the *x*-*z* and *x*-*y* plane cross-sections of the diatom 3D tomography, which were reconstructed from the TXM raw data (shown in Supplementary Movie S3). Significant blurring can be observed in the reconstruction and the corresponding sinogram. The quality of the tomographic reconstruction is highly affected by the positional jitters in the raw images, which are caused by small mechanical or thermal instabilities in the high-resolution tomography system. Figure 7(c) shows the reconstruction and the corresponding sinogram that was aligned using the simple cross-correlation method. Significant distortions in the specimen reconstruction can be observed, particularly in the region containing the gold particle that was attached to the microfossil, as indicated by the yellow arrow in Fig. 7(c). Figure 7(d–g) show the reconstruction sections and the corresponding sinograms that were aligned using the JI-Faproma method (The well-aligned image dataset is shown in Supplementary Movie S3). It can be observed from Fig. 7(d–g) (yellow arrows) that the reconstruction converges to its optimal value after only 5 iterations, thus demonstrating the ability of the JI-Faproma method to achieve fast convergence in a real X-ray nano-tomography case.

**Figure 8:**
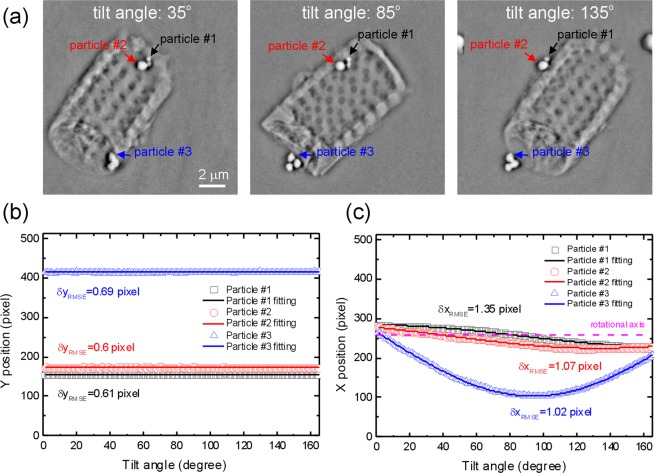
Alignment accuracy of the JI-Faproma method evaluated by marker tracking method. (**a**) JI-Faproma aligned diatom TXM images obtained from different tilt angles. (**b**) Relative corrected vertical positions of the three nanoparticle markers corresponding to each tilt angle. The vertical RMSEs between the aligned positions and linear fits for the three nanoparticles are 0.61, 0.6, and 0.69 pixels, respectively. (**c**) Horizontal RMSEs between the aligned positions and fitted sinusoidal curves for the three nanoparticles is given by 1.35, 1.07, and 1.02 pixels, which corresponds to a shift of 40, 32, and 31 nm in real space.

The alignment accuracy of the JI-Faproma method was evaluated by attaching and accurately positioning a few small round gold nanoparticles on the diatom using a particle tracking software (the MTrack2 plugin module in ImageJ). The gold nanoparticles were freely supported and background correction was applied to the images to ensure that the position of each nanoparticle, determined by its center of mass, is theoretically accurate. In order to validate the alignment accuracy of each projection using the JI-Faproma method, linear and sinusoidal functions were fit to the vertical and horizontal nanoparticle positions to obtain the deviation of the aligned nanoparticles from their theoretical positions. Three small nanoparticles of different sizes were evaluated as shown in Fig. 8(a). Figure 8(b) depicts the relative corrected vertical positions of the three nanoparticles corresponding to each tilt angle. The vertical RMSEs between the aligned positions and linear fits for the three nanoparticles are 0.61, 0.6, and 0.69 pixels, respectively. A maximum deviation of 0.69 pixels corresponds to a 21 nm displacement in real space, which is much lower than the 60-nm spatial resolution of the TXM. The high vertical alignment accuracy demonstrates that the JI-Faproma algorithm corrects in-plane rotational errors with high accuracy. Figure 8(c) shows that the horizontal RMSEs between the aligned positions and fitted sinusoidal curves for the three nanoparticles is given by 1.35, 1.07, and 1.02 pixels, which corresponds to a shift of 40, 32, and 31 nm in real space, respectively. The small RMSEs along the horizontal and vertical directions indicate that there is a negligible shift during the alignment process, which is well below the spatial resolution limit of the TXM instrument. This demonstrates that the proposed JI-Faproma method achieves reliable marker-free image registration and is comparable to the highly accurate marker-based particle-tracking method.

## Discussion

In classical projection matching methods, the iterative reconstruction is the most time-consuming process. Ignoring the computation time required for other processes, the total processing time of the classical projection matching method can be expressed as *t*_pm_ α *N*_iter-recon_*t_recon_*L* *N*_pm_, where *N*_iter-recon_ is the number of iterations required for reconstruction, *t*_recon_ is the processing time for once reconstruction, *L* is the total number of reconstruction layers, and *N*_pm_ represents the number of iterations required for projection matching. The total processing time of the Faproma method is lower than that of the classical projection matching method, and is given by *t*_Faprma_ α *N*_iter-recon_*t_recon_* *N*_Faproma_ + *t*_vertical_, where *N*_Faproma_ represents the number of iterations required for Faproma, *t*_vertical_ is the total processing time required to compute the vertical shift alignment using the common-line method. However, the total processing time of the JI-Faproma method can be further reduced and is given by *t*_JI-Faprma_ α *N*_iter-recon_*t_recon_ + *t*_vertical_. This clearly shows that the total processing time of the JI-Faproma method is almost an order of magnitude less than that of the Faproma method as shown in Fig. 6. Furthermore, the total processing time of the JI-Faproma method is less than that of the classical projection matching method by approximately three to four orders of magnitude, and is especially advantageous when processing large images.

Table 1 shows a comparison between the total processing times required by the classical projection matching, Faproma, and JI-Faproma methods evaluated on the ideal test phantom described previously and the real X-ray nano-tomography case. The test phantom dataset consisted of 181 projection images, each with a pixel number of 511 × 511. The total processing time of the classical projection matching method as shown in Table 1(a) is estimated as *t*_pm_ > 3832500 s, where *N*_iter-recon_ = 50, *t*_recon_ = 3 s, *L* = 511, and *N*_pm_ = 50. The time taken to process once reconstruction using the ML-EM iterative reconstruction algorithm was *t*_recon_ = 3 s, which was computed using a tabletop workstation computer (Z600, HP) equipped with two 2.4 GHz processors and a graphics processing unit (GPU, NVIDIA Quadro K6000), Table 1(b) shows that by using the Faproma method, the total processing time for the same dataset can be reduced to 516 s. The JI-Faproma method further reduces the total processing time to 127 s and is illustrated in Table 1(c). The total processing time of the JI-Faproma algorithm was also evaluated for the alignment of a real data set obtained using the TXM, and is shown in Figs. 7 and 8. The total alignment processing time was approximately 2 minutes by using the tabletop workstation computer. Thus, the JI-Faproma algorithm facilitates marker-free alignment in an acceptable time frame and can be easily implemented on most desktop computers.

**Table 1:** Comparison between the total processing times required by the (a) classical projection matching, (b) Faproma, and (c) JI-Faproma methods evaluated on the ideal test phantom and (d) real X-ray nano-tomography case.

Case	(a) Test phantom	(b) Test phantom	(c) Test phantom	(d) TXM data
Alignment method	Classical PM	Faproma	JI-Faproma	JI-Faproma
Data size	511 × 511 pixels (181 images)	511 × 511 pixels (181 images)	511 × 511 pixels (181 images)	512 × 512 pixels (171 images)
Processing time for the in-plane rotational and vertical error correction	NA	78 s (1 iteration, measured)	78 s (1 iteration, measured)	74 s (7 iteration, measured)
Processing time for the horizontal error correction	>3832500 s (50 × 50 iteration, estimated)	428 s (50 × 10 iteration, measured)	49 s (15 iteration, measured)	49 s (15 iteration, measured)
Total processing time	>3832500 s	516 s	127 s	123 s

In this study, a simple version of the Faproma algorithm was implemented in Matlab (Matlab R2014b, The MathWorks, Inc.) to demonstrate the benefit of this technique. Further optimization of the algorithm could lead to higher processing speeds, particularly with the implementation of robust parallel processing/computing. The JI-Faproma alignment algorithm offers the added advantage of substantially reducing the hardware requirements for both data processing and tomographic acquisition, and can also be easily integrated into commercial tomographic reconstruction software.

## Supplementary information

supplementary information.

supplementary information2.

supplementary information3.

supplementary information4.
