# Supplementary material for: Joint Iterative Fast Projection Matching for Fully Automatic Marker-free Alignment of Nano-tomography Reconstructions
Source: Sci Rep. 2020 Apr 30;10:7330. doi: 10.1038/s41598-020-62949-1 (PMC7192921; doi:10.1038/s41598-020-62949-1)
Supplement: Supplementary file 1 — supplementary information. [file 41598_2020_62949_MOESM1_ESM.docx]

Supplementary Information

**Joint Iterative Fast Projection Matching for Fully Automatic Marker-free Alignment of Nano-tomography Reconstructions**

Chun-Chieh Wang^*^

National Synchrotron Radiation Research Center, 30076Hsinchu, Taiwan.

**CORRESPONDING AUTHOR**

Chun-Chieh Wang (E-mail: [wang.jay@nsrrc.org.tw](mailto:song@nsrrc.org.tw))


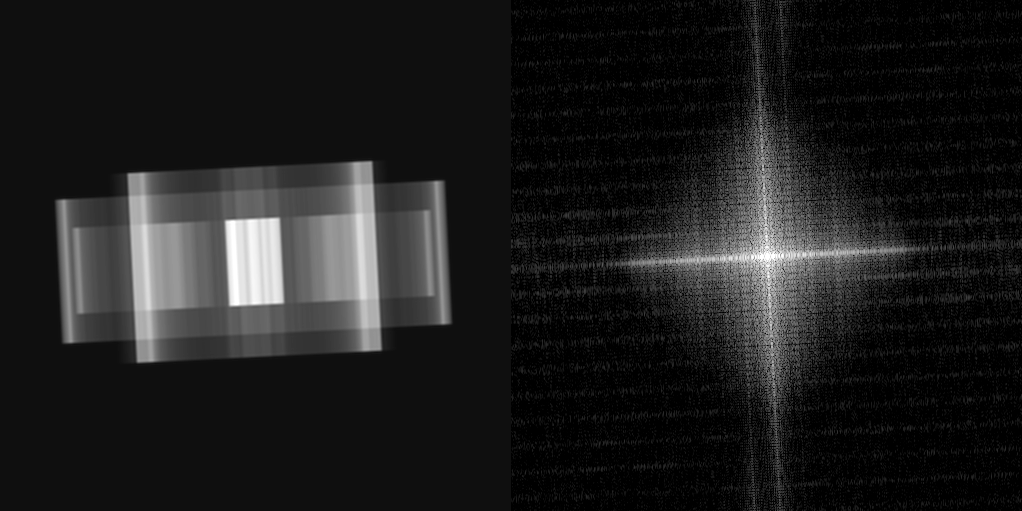


**Supplementary Movie S1**. Raw projections and their corresponding 2D Fourier Transforms of 3D test phantom with random errors in the* *, *y*, and *x* dimensions obtained from different tilt angles.


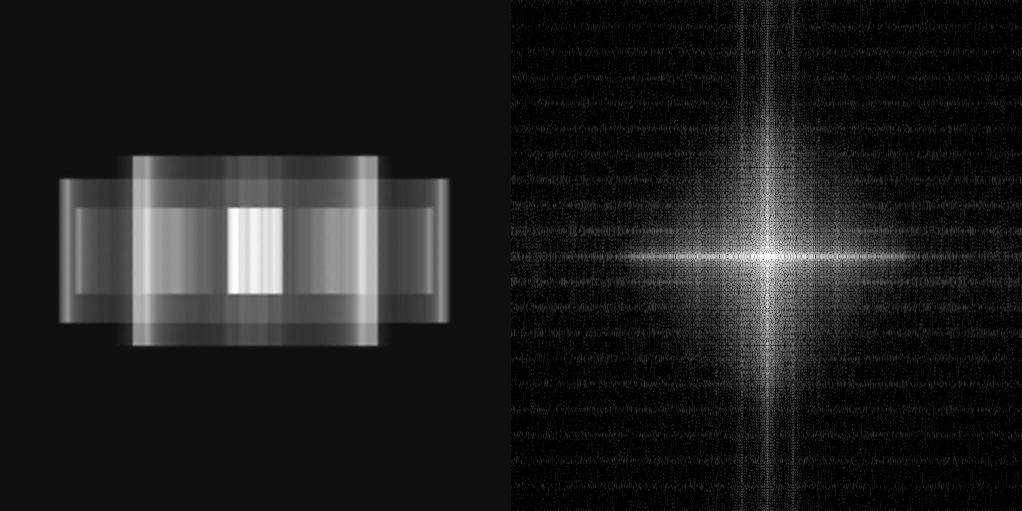


**Supplementary Movie S2**. Fine-aligned projections and their corresponding 2D Fourier Transforms obtained from different tilt angles after the image alignment process of the J-I Faproma.


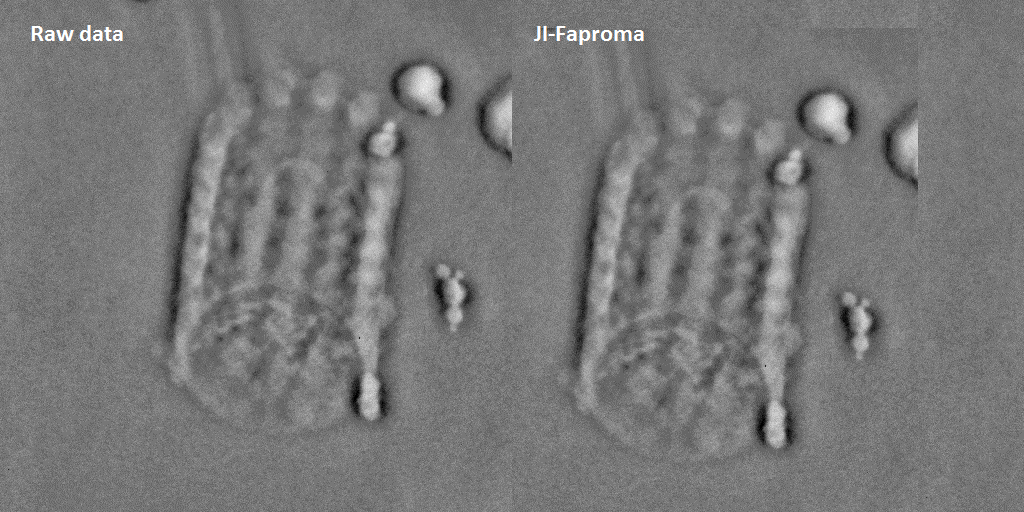


**Supplementary Movie S3**. Projections of raw and JI-Faproma aligned data sets obtained from different tilt angles.
